# Post-stroke BDNF Concentration Changes Following Physical Exercise: A Systematic Review

**DOI:** 10.3389/fneur.2018.00637

**Published:** 2018-08-28

**Authors:** Carolina C. Alcantara, Luisa F. García-Salazar, Marcela A. Silva-Couto, Gabriela L. Santos, Darcy S. Reisman, Thiago L. Russo

**Affiliations:** ^1^Laboratory of Neurological Physiotherapy Research, Physical Therapy Department, Federal University of São Carlos, São Carlos, Brazil; ^2^Escuela de Medicina y Ciencias de la Salud, GI Ciencias de la Rehabilitación, Universidad del Rosario, Bogotá, Colombia; ^3^Department of Physical Therapy, University of Delaware, Newark, DE, United States

**Keywords:** Brain-derived neurotrophic factor, neuroplasticity, exercise, rehabilitation, stroke

## Abstract

**Background:** Research over the last two decades has highlighted the critical role of Brain-derived neurotrophic factor (BDNF) in brain neuroplasticity. Studies suggest that physical exercise may have a positive impact on the release of BDNF and therefore, brain plasticity. These results in animal and human studies have potential implications for the recovery from damage to the brain and for interventions that aim to facilitate neuroplasticity and, therefore, the rehabilitation process.

**Purpose:** The aim of this study was to carry out a systematic review of the literature investigating how aerobic exercises and functional task training influence BDNF concentrations post-stroke in humans and animal models.

**Data Sources:** Searches were conducted in PubMed (via National Library of Medicine), SCOPUS (Elsevier), CINAHL with Full Text (EBSCO), MEDLINE 1946—present with daily updates (Ovid) and Cochrane.

**Study Selection:** All of the database searches were limited to the period from January, 2004 to May, 2017.

**Data Extraction:** Two reviewers extracted study details and data. The methodological quality of the studies that used animal models was assessed using the ARRIVE Guidelines, and the study that evaluated human BDNF was assessed using the PEDro Scale.

**Data Synthesis:** Twenty-one articles were included in this review. BDNF measurements were performed systemically (serum/plasma) or locally (central nervous system). Only one study evaluated human BDNF concentrations following physical exercise, while 20 studies were experimental studies using a stroke model in animals. A wide variation was observed in the training protocol between studies, although treadmill walking was the most common type of intervention among the studies. Studies were of variable quality: the studies that used animal models scored from 8/20 to 15/20 according to the ARRIVE Guidelines. The only study that evaluated human subjects scored 5/10 according to the PEDro scale and, which indicates a quality classified as “fair”.

**Conclusions:** The results of the current systematic review suggest that aerobic exercise promotes changes in central BDNF concentrations post-stroke. On the other hand, BDNF responses following functional exercises, such as reaching training and Constraint Induced Movement Therapy (CIMT), seem to be still controversial. Given the lack of studies evaluating post-stroke BDNF concentration following physical exercise in humans, these conclusions are based on animal work.

## Introduction

Research over the last 10 years has demonstrated that Brain-derived neurotrophic factor (BDNF) plays an important role in brain plasticity in the intact brain (1, 2), as well as after central nervous system (CNS) damage (3, 4). BDNF is a member of the neurotrophin family, known for its role in neuronal proliferation, survival and differentiation (1). Along with its receptor tyrosine kinase, this neurotrophin is largely distributed throughout the healthy human brain (5, 6).

The role of BDNF after stroke has been highlighted in many studies (3, 4, 7–10). Its action is related not only to the induction of anti-apoptotic mechanisms, reducing the size of the lesion, but also to secondary neuronal death (3) Furthermore, motor learning post-stroke has been related to increases in BDNF concentrations in the cortex (10), which may accomplish cortical map reorganization through synaptogenesis, enhanced dendritic spine formation and ramification, thereby contributing in many ways to neuronal plasticity post-stroke (3, 4, 9).

Given the evidence linking BDNF and brain plasticity, research advancements have been made aiming to understand the response of BDNF levels to physical exercise training and how these changes would mediate the beneficial effects of exercise on learning (8, 11, 12). This systematic review specifically emphasized studies that examined exercise intervention, not exercise as a priming (e.g.,-single session, before and after exercise measurements only). Converging results available in the literature suggest that aerobic exercise training may lead to an increase in BDNF concentrations in neurologically intact humans (13). Thus, using aerobic training as an intervention to optimize neuroplasticity and recovery in patients post-stroke has gained considerable interest (10). Although there is still a lack of studies evaluating BDNF concentrations following exercise in subjects post-stroke, evidence based on stroke-induced animal models suggests a relationship between aerobic exercise training and an increase in BDNF concentrations (14–16) Furthermore, recent studies have also measured BDNF concentrations following functional task training in stroke-induced animals models, such as skilled reach training, in order to further clarify the mechanisms by which these interventions would induce recovery (17, 18).

However, given the controversial results between studies, the relationship between exercise training (aerobic and/or functional task training) and BDNF levels post-stroke has not been fully elucidated. Although previous literature reviews have addressed the effects of aerobic exercise on neuroplasticity in general after stroke (7, 8) the focus of the current review is on the role of BDNF in the physical exercise response, either aerobic and/or functional task training, in human or animal models of stroke. Thus, the primary objective of this study was to carry out a systematic review of literature investigating the effects of aerobic exercise and functional task training on BDNF concentrations in animals or humans post-stroke. The secondary objective of this review was to analyze the methodological quality of selected studies.

## Methods

### Search methods

Searches were conducted in May 2017 using the following electronic databases: PubMed (via National Library of Medicine), SCOPUS (Elsevier), CINAHL with Full Text (EBSCO), MEDLINE 1946—present with daily updates (Ovid) and Cochrane. The following MeSH headings or keywords were used: “Brain Derived Neurotrophic Factor” OR “BDNF” AND “stroke” OR “cerebrovascular accident”. All of the database searches were limited to the period from January, 2004 to May, 2017.

For the search and study selection, the following inclusion criteria were used: (1) the primary objective of the study was to evaluate the effect of physical exercise (aerobic exercise and/or functional task training) on BDNF concentrations in humans or animals and (2) full papers published in English. Studies were excluded in the following sequence: any article that did not involve BDNF; studies that evaluated BDNF levels in other conditions, rather than stroke; studies that had primary objectives other than stated in #1 above (e.g.,-validation of an analysis method); and interventions that did not involve physical exercise. Furthermore, reviews, case studies, commentaries, letters and guidelines were excluded.

### Study selection

To identify potentially eligible articles, two reviewers (CCA and LFG) independently assessed the titles and abstracts obtained from the electronic search according to inclusion and exclusion criteria. After this first selection, full-length articles were read by the same reviewers in order to define which articles met all the inclusion criteria. However, if there was a disagreement between two reviewers, a third reviewer was consulted. If the full text was not available, the authors were contacted and asked if they could send their research papers. Furthermore, the authors verified the reference lists from each included paper to check if there were relevant publications (not included in the initial search) and manually searched for them. To systematize and organize the search and data extraction, the *State of the Art through Systematic Review* (StArt) (Available from: http://lapes.dc.ufscar.br/tools/start_tool) was used.

### Data extraction and quality assessment

A standardized electronic data extraction form was developed to obtain key information relevant to this review. Data extraction for each article were: sample size, characteristics of injury (type—mechanism and time post-injury), species evaluated, measuring technique, site and type of tissue (whether systemically or locally measured, and in which tissue, for example, serum, plasma, brain, muscle, etc.), protocol of exercise, instruments of assessment, time between measurements and main results. The last item (main results) involves the change in BDNF concentrations (increased, decreased or not changed) and if there was any correlation between change concentrations and motor impairment.

The methodological quality of the included studies that used animal models was assessed using the ARRIVE Guidelines. It consists of 20 items, such as the number and specific characteristics of animals used and the experimental, statistical and analytical methods (including details of methods used to reduce bias such as randomization and blinding). The methodological quality of the included study that evaluated human BDNF (19) was assessed using the Physiotherapy Evidence Database (PEDro) Scale (20). The checklist consisted of 11 items related to the study design, reporting eligibility criteria, between-group statistical comparisons and variability measures. In accordance with the total score from the PEDro Scale, the article can be classified into: excellent (10–11), good (6–9), fair (4–5), and poor (less than 4).

## Results

**Figure 1:**
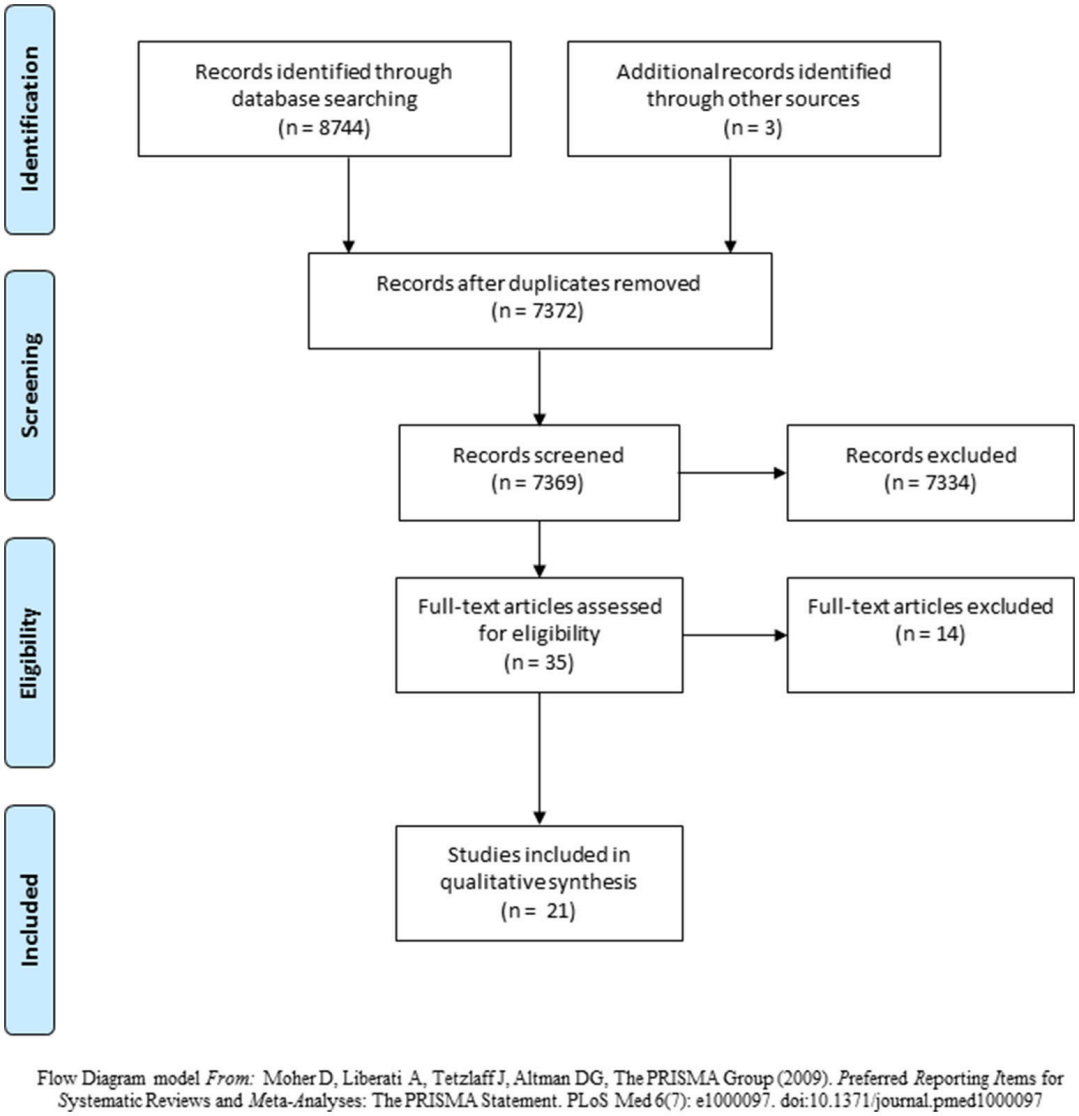
Flowchart for literature search.

A total of 8744 articles were identified through database searches (Cochrane, *n* = 1,674; SCOPUS, *n* = 954; CINAHL, *n* = 778; PubMed, *n* = 1,995; MEDLINE, *n* = 3,343). After the duplicates were removed, 7372 articles were listed, although 3 of them were mentioned as “unclassified articles”. Therefore, the titles and abstracts of 7369 articles were screened. Out of these, 7334 articles were excluded primarily because they did not involve BDNF measurements or were evaluated BDNF levels in other conditions, rather than stroke. Finally, 35 full-text manuscripts were read. Another 3 articles from the references were added to the search. In the end, only 21 articles fulfilled all of the inclusion criteria (Figure 1).

Table 1 presents information regarding the included studies that evaluated the effects of physical activity on the BDNF levels. The table is divided into three sections, according to the type of exercise investigated in the study: (1) aerobic exercise; (2) functional training; (3) both (aerobic exercise and functional training). Aerobic exercise (mainly treadmill training) was the most common type of intervention among the studies and most of them showed an increase in BDNF concentration after this intervention. Only one study included in this review was completed in human subjects (19) 0.2 (5). Therefore, most of the understanding of changes in the BDNF concentration with exercise arises from animal studies. Generally speaking, this systematic review showed that central BDNF responses following non-aerobic exercise training in the animal model of stroke are still controversial, whereas aerobic exercise training appears to promote changes in central BDNF concentrations post-experimental stroke in animals.

**Table 1:** Methodological characteristics and main results of studies that evaluated the effects of physical activity on the BDNF levels post-stroke.

**Article**	**Sample size**	**Characteristics of Injury**		**Characteristics of the sample**	**Methodology of measurement**	**Site of measurement**	**Protocol of exercise**	**Instruments of motor assessment**	**Time between measurements**	**Main results**
		**Type (mechanism)**	**Time post-injury**							
**AEROBIC EXERCISE**										
(14)	Exercice (*n* = 18) Non-exercice (*n* = 17) Sham (*n* = 12)	Ischemic Middle Cerebral Artery Occlusion	4 days	Sprague-Dawley Rats	Western Blotting Immunohistochemistry	Systemic: — — Central: ipsilateral and contralateral	Treadmill: For 12 days at 30 min per day. Initial velocity was 10 m/min and velocity progression was 5 m/min per week.	Motor behavior index	Behavioral test: 2, 9, 16 days post-stroke BDNF: 16 days post stroke	Treadmill exercise for 2 weeks promoted motor function and changed the expressions of BDNF and trkB proteins. The expressions of BDNF and full-length trkB were increased in the contralateral hemisphere.
(21)	Sedentary group (*n* = 8) Fast walk for 30 min in motorized wheels (*n* = 12) Fast walk for 60 min in motorized wheels (*n* = 11) Run for 30 min in motorized wheels (*n* = 10) 12 h voluntary run (*n* = 11)	Ischemic (endothelin-1)	4 days	Sprague-Dawley Rats	ELISA	Systemic: — — Central: ipsilateral/ contralateral hippocampus and córtex	Motorized running wheel: from 4 m/min for 5 min to a walking pace of 9 m/min for 20 min over 7 days. Voluntary running: began at 5 min progressing to 60 min over 7 days.	— — — — — –	2 weeks post-intervention	BDNF levels were enhanced by both the ischemia and either a single 30 min walk or 12 h voluntary run.
(22)	Sedentary (*n* = 10) Motorized running wheels: 0 min (*n* = 10) 30 min (*n* = 6) 60 min (*n* = 6) 120 min (*n* = 5) Voluntary running wheels: 0 min (*n* = 10) 30 min (*n* = 6) 60 min (*n* = 6) 120 min (*n* = 5)	Ischemic (endothelin-1)	4 days	Sprague-Dawley Rats	ELISA	Systemic: — — Central: Ipsilateral/ contralateral hippocampus and córtex	Voluntary running wheel: progressed from 5 to 60 min over 7 days. Motorized running wheel: progressed from 4 m/min for 5 min to 9 m/min for 20 min over 7 days.	— — — — — –	2 weeks post-intervention	BDNF increased after both motorized and voluntary training. However, after motorized training, a peak was not maintained over time. After voluntary training, BDNF increasing was maintained for a longer time.
(23)	Involuntary exercise (I-Ex, *n* = 14) Voluntary wheel exercise group (V-Ex, *n* = 14) Forced treadmill exercise group (F-Ex, *n* = 15) Control group (Con, *n* = 14)	Ischemic Middle Cerebral Artery Occlusion	24 hours	Sprague-Dawley Rats	ELISA	Systemic: — – Central: ipsilateral striatum and motor cortex, and hippocampus	V-Ex rats: 23 h voluntary wheel running for 7 days F-Ex rats: motor-driven treadmill at a speed of 20 m/min with a slope of 0° for a total of 30 min every day, for 7 days I-Ex: 30 minutes FES every day, for 7 days	De Ryck's behavioral Test	Behavioral test: daily during the 7-day intervention period (I1–I7) and repeated for three times after everyday intervention BDNF: post-intervention	After training, Vex had significant higher behavioral test score than I-Ex, F-Ex, and Con. Both V-Ex and I-Ex had higher hippocampal BDNF concentration than F-Ex and Con. Besides, I-Ex had significantly higher striatal and cortical BDNF concentrations than F-Ex and Con
(24)	Control group (*n* = 17) Exercise Treadmill Group (*n* = 17)	Hemorrhagic (heparinate bacterial collagenase)	7–14 days	C57/BL6 Rats	Immunohistochemistry	Systemic: — — Central: both hemispheres	Treadmill running: Intensity: from 40 m/min per 5 min to 80 m/min per 10 min for 10 days	— — — — — –	7 and 14 days post-intervention	BDNF-TrKB was increased 7 days post-training for both groups and returned to initial levels in control 14 days post-injury
(25)	Total = 32 SED control SED stroke EX control Ex stroke	Ischemic (phototrombotic)	14 days	Wistar rats	ELISA Western Blotting Immunohistochemistry	Systemic: — — Central: ipsilateral hippocampus, striatum and cortical area	Treadmill training: 0.3 m/s for 30 minutes by 7 days	—	Stroke: 15 days post-intervention Control: 7 days post-intervention	Cortex was the most responsive area after exercise. Exercise resulted in a comparable increase in the production of mature BDNF in intact and stroke rats but increased proBDNF levels only in intact rats
(15)	MCAO group (*n* = 12) MCAO+Ex group (*n* = 13)	Transient Middle Cerebral Artery Occlusion (30 min)	5 days post stroke	Sprague-Dawley Rats	Western Blotting	Systemic: — — Central: ipsilateral cortex and striatum	Treadmill training 30 min 5 days per week Duration: 5th-28th day post-stroke	Rotarod (behavioral test)	Behavioral test: 3, 7, 14, 21, 28, 35, 42, 49, 56, and 63 days post stroke BDNF: 91 days post-stroke (63 days after the end of training)	Exercise improved functional recovery and increased BDNF levels in cortex and striatum.
(16)	sed VO (*n* = 6) sed sham (*n* = 6) trained VO (*n* = 6) trained sham (*n* = 6)	Left Common Carotid Artery Occlusion	1–7 days	Wistar rats	Western Blotting	Systemic: — — Central: ipsilateral and contralateral motor areas	Treadmill training (7 days, 30 min/day, 18 m/min):	— — — — — –	BDNF: post-intervention	Treadmill training increased BDNF levels in the contralateral hemisphere.
(19)	Control group (*n* = 15) Study group (*n* = 15)	Ischemic	3–18 months	Human	ELISA	Systemic: serum Central: — —	G1: Conventional physical therapy (Stretching, facilitation for weak muscles, strengthening, posture control, balance, gait and functional training) G2: Conventional physical therapy + Bicycle ergometer (40–45 min) Protocol: 3 times/week by 3 weeks	— — — — — –	Pre and post 8 weeks training	G2 showed higher BDNF levels compared to G1 post-training. There were correlation between BDNF concentration and cognitive function post-training.
(26)	pMCAO group (*n* = 20), pMCAO + Ex group (*n* = 15) sham-operated group (*n* = 20)	Right Middle Cerebral Artery Occlusion	3–19 days	Sprague-Dawley Rats	Western Blotting	Systemic: — — Central: brain tissue	Treadmill training: 10 m/min for 20 min per day in the first 2 days, then 15 m/min for 30 min per day in the following 14 days	mNSS	Neurological Function: 24 h, on day 3, 8, 12 and 19 after lesion BDNF: post-surgery and intervention	The mNSS in pMCAO + Ex group was lower than that in pMCAO group on day 19 post-MCAO. The protein expressions levels of BDNF was downregulated after cerebral ischemia and upregulated after treadmill exercise.
(27)	sham-operation group (*n* = 10) sham-operation + exercise group (*n* = 10) ischemia-induction group (*n* = 10) ischemia-induction + exercise group (*n* = 10)	Common Carotid Arteries Occlusion	1 day and 2 weeks	Gerbil	Western Blotting	Systemic: — — Central: Hippocampus	Treadmill training: 30 min, once a day for 2 weeks, at a speed of 2 m/min for the first 5 min, 5 m/min for the following 5 min, and 8 m/min for the last 20 min	— — — — — –	BDNF: post-surgery and intervention	BDNF expression was increased by the induction of ischemia, while treadmill exercise further increased BDNF expression in the ischemic gerbils.
(28)	Control Group (*n* = 15) Low Training (*n* = 15) Gradually Increasing (*n* = 15) High Training (*n* = 15)	Middle Cerebral Artery Occlusion	1–7 days	Sprague-Dawley	ELISA	Systemic: — – Central: hippocampus, striatum, and sensorimotor cortex	Treadmill training: for 7 days Low Training - 30 min with a 10-min rest between 10 min of running section at a velocity of 5 m/min High Training: at 26 m/min with the same training and rest regimens Gradually Training: from 5 m/min on the 1st day (D1) up to 26 m/min on the last day (D7)	Longa's test De Ryck behavioral test	Behavioral Tests: 24 h post-lesion, and daily BDNF: pre training, 2 h after last training session (8 days post-stroke)	Hippocampal BDNF concentrations were significantly higher than in both the striatum and cortex for all groups. Gradually intensity rats showed the highest BDNF levels in the hippocampus and striatum. BDNF levels in Low Intensity and High Intensity rats were significantly higher in the hippocampus and striatum than control rats.
(29)	Sham group (*n* = 14) Ischemia group (*n* = 7) Sedentary group (SD4, *n* = 14) One week treadmill (TR1, *n* = 14) Four weeks treadmill (TR4, *n* = 14)	Common carotid arteries occlusion	5 days - 4 weeks post-stroke	Mongolian gerbils	Immunohistochemistry Western Blotting	Systemic: — — Central: hippocampus, CA1 region and dentate gyrus	Treadmill training: 30 min/day, 5 days/week for 1 (TR1) or 4 (TR4) consecutive weeks, at speed of 5 m/min for the first 5 min, 7 m/min for the next 5 min, and 10 m/min for the last 20 min with 0° inclination.	— — — — — –	BDNF: 5 days after injury, 1 and 4 weeks from 5 days post-ischemia.	No BDNF immunoreactivity was observed in sham group; For SD4 group, it was strong expressed in CA1 and DG. For TR4, the density of BDNF of CA1 region is similar to the SD4 groups, and higher in the DG compared with SD4. Regarding to protein levels (CA1 and DG), all groups presented higher values compared to sham, SD4 showed higher lower levels only in DG compared to TR groups, and TR4 had higher levels only DG compared to TR1. For protein levels in hippocampus, TR groups presented higher levels compared to sham group, and TR4 groups also showed higher levels compared to SD4.
(30)	Sham group (*n* = 7) Non-exercise group (NE, *n* = 12) Early exercise group (Early, *n* = 7) Late exercise group (Later, *n* = 7)	Right external carotid artery occlusion with injection polystyrene into common carotid artery	1–22 days	Sprague-Dawley Rats	ELISA	Systemic: — — Central: right hippocampus	Treadmill training: 15 m/min for 30 min every day during 1 week.	— — — — — –	BDNF: before injection, at 1, 8, 15, and 22 day after injection	Compared to the moment before injection, the early and late groups presented higher BDNF levels at 7 and 15 days, respectively.
(31)	Sham control (SC, *n* = 11) Sham exercise (SE, *n* = 11) ICH control (IC, *n* = 14) ICH exercise (IE, *n* = 14)	Hemorrhagic (collagenase type IV)	1–15 days	Wistar rats	Western Blotting	Systemic: — — Central: ipsilateral and contralateral motor cortex	Treadmill training: 30 minutes, once a day for 11 consecutive days (from day 4 to day 14 after injury), at a speed of 9 m/min	Motor deficit score, Beam-walking test Cylinder test	Behavioral Tests: 1, 3, 7, 10, and 15 days after injury (motor deficit and beam-walking test) and 1 day prior surgery an at 3, 7, and 15 days after surgery (cylinder test) BDNF: at 15 days after injury	Motor function of exercise group with injury (IE) improved in all behavior tests compared with exercise control group (IC). TrkB expression levels increased in IE group compared to IC, however, no differences in BDNF expression levels was found between groups.
**FUNCTIONAL TRAINING**										
(18)	Control Stroke (*n* = 11) Control Sham (*n* = 12) FUMT Stroke (*n* = 11) FUMT Sham (*n* = 11)	Ischemic (endothelin-1)	1–21 days	Sprague-Dawley Rats	Immunohistochemistry	Systemic: — — Central: both hemispheres	CIMT during 30 min daily	TFP, VFP, forelimb postural reflex test, Schallert cylinder test, Horizontal ladder test	1, 3, 6, 10, 14, 18, and 21 days post-injury	CIMT accelerated the functional recovery of the limb when compared to the control, but did not affect the expression of BDNF in the hemisphere ipsilateral to the lesion.
(32)	Total = 20 Control group (CON): 1 week post-stroke (CON1) 4 weeks post-stroke (CON4) Experimental group (SRT - skilled reach training) 1 week post-stroke (SRT1) 4 weeks post-stroke (SRT4)	Hemorrhagic (collagenase type VII)	1 and 4 weeks	Sprague-Dawley Rats	Western Blotting	Systemic: — — Central: brain tissue	Skilled reach training (plexiglass chamber) 15 min 6 days/week for 1 or 4 weeks	Skilled ladder rung walking test	1 and 4 weeks post-training	BDNF expression increased after 4 weeks of training comparing to pre-training and control group. Functional task also improved on 4 weeks experimental group, with better performance compared to control group.
(17)	Hemorrhage and non-treated group (ICH group: *n* = 9) Early-CIMT group (E-CIMT, *n* = 8) Late-CIMT group (L-CIMT, *n* = 6) Sham-operated group (sham-group, *n* = 6)	Hemorrhagic (collagenase type IV)	8–28 days	Wistar rats	Immunohistochemistry Gene expression (PCR)	Systemic: — — Central: ipsilateral and contralateral sensorimotor cortex	Forcing rats to use the affected forelimb in all daily activities for 7 days starting either 1 day (early CIMT) or 17 days (late CIMT) after the lesion.	Skilled reaching test Horizontal ladder stepping test	Behavioral Tests: 10–12 and 26–28 after the lesion (reaching) and on days 12 and 28 after the lesion (ladder) BDNF: after early CIMT (day 8) and later CIMT (day 24).	Early-CIMT improved reaching and stepping function of impaired forelimb after injury, but late-CIMT did not. Early-CIMT induced an increase in ipsilesional levels of BNDF, however, did not change levels in contralesional. Later-CIMT failed to induce changes in the BDNF levels.
(33)	Sham-operated (Sham, D14: *n* = 6, D29: *n* = 6) No treatment (ICH, D14: *n* = 6, D29: *n* = 7) ICH + Acrobatic Training (ICH+AT, D14: *n* = 6, D29: *n* = 6)	Hemorrhagic (collagenase type IV)	1–28 days	Wistar rats	Gene expression (PCR)	Systemic: — — Central: somatosensory cortex	Each of the 5 acrobatic tasks (rope ladder, grating platform, rope, parallel bar, barrier) was performed spontaneously with 4 trials each day 4–28 days after surgery.	Forepaw Grasping, Modified Forelimb Placing, Postural Instability Test	Behavioral Tests: at day 1, 3, 7, 14, 12, and 28 after surgery. BDNF: At 14 and 29 days after surgery.	Motor skills training after ICH enhanced the forelimb sensorimotor function. At 14 days after surgery, the BDNF mRNA expression level was downregulated in the ipsilesional cortex by ICH, and it was not upregulated by acrobatic training. At 29 days, the ICH+AT group had higher mRNA expression levels of BDNF in the ipsilesional sensorimotor cortex than the sham group.
**AEROBIC EXERCISE** + **FUNCTIONAL TRAINING**										
(34)	No rehab (*n* = 7) Reach (*n* = 7) Run (*n* = 7) Run/Reach (*n* = 8)	Ischemic (endothelin-1)	5 days	Sprague-Dawley Rats	Immunohistochemistry Gene expression (PCR)	Systemic: — — Central: ipsilateral and contralateral cingulate cortices, contra- lateral primary motor cortex and contralateral sensory cortex	Reaching training associated or not with previous aerobic training (running) Duration: 30-120 min/day Over 5 weeks	Skilled reaching test (staircase apparatus), forelimb asymmetry test (Plexigas cylinder), Ladder rung walking test	Functional tests: 2 days prior to stroke, 3 days post-stroke and 2, 3, 4, and 5 weeks after stroke BDNF: 3 days following the last rehabilitation session	Aerobic exercise followed by reaching training improved reaching skill. There was no group effect on expression of BDNF.
(35)	Total = 30 Control (CON) Skilled reach training (SC) Treadmill exercise (TE)	Left Middle Cerebral Artery Occlusion	2 weeks	Sprague-Dawley Rats	Western Blotting	Systemic: — — Central: brain tissue	Skilled reach training (plexiglass chamber): 30 min 6 days/week for 2 weeks Treadmill training: not described.	— — — — — –	BDNF: 2 weeks after surgery	BDNF expression in SC and TE groups were higher compared to CON group, however, no differences between SC and TE groups were observed.

### Methodological characteristics

The included studies have a longitudinal design, i.e., BDNF concentrations were assessed after conducting an exercise training program. A wide variation was observed in exercise training protocols between studies regarding duration, intensity and frequency of treatment sessions and the time post-stroke at which the training started (Table 1). Two main types of exercise were addressed in the studies included in this review: (1) Aerobic exercise; and/or (2) Functional training.

#### Aerobic exercise

Fifteen studies evaluated the effect of aerobic exercise training (Treadmill/ Bicycle ergometer/Motorized running wheel) on BDNF concentrations (14–16,, 19, 21–31, 35). Only one study evaluated systemic BDNF concentration following aerobic exercise training in humans post-stroke (19), while all others evaluated central BDNF concentration in animal models of stroke. The sample size per experimental group varied from 4 to 20. The methods used to measure the BDNF concentration include ELISA, Immunohistochemistry and Western Blotting. The time at which the BDNF measurement was performed varied from 8 days post-injury until 91 days post-injury among the studies/experimental groups.

#### Functional training

Among all 21 studies included in this review, 6 studies evaluated the effects of functional training on BDNF concentration (17, 18, 32–35) All of them were performed using an experimental stroke model in rats. Functional training such as reaching tasks, Constraint-Induced Movement Therapy (CIMT) and acrobatic training were performed in those studies. One of them compared BDNF concentrations after isolated reaching training to those following a reaching training preceded by aerobic exercise (34). The sample size per experimental group varied from 5 to 12 among these 4 studies. The methods used to measure the BDNF concentration include Immunohistochemistry, Western Blotting and Gene expression (PCR). The time at which the BDNF measurement was performed varied from, 7 days post-injury to 43 days post-injury among the studies/experimental groups.

### Methodological quality of studies

All articles that used animal models were scored using the ARRIVE Guidelines. The score ranged from 8/20 to 15/20. All these articles provided, for example, an accurate summary of the background in the Abstract, provided details of the animals used, and described the outcomes and estimation (reported the results for each analysis carried out with a measure of precision). None of them reported regarding adverse effects or described any modifications to the experimental protocols made to reduce adverse events. Besides, none of them explained how the number of animals was decided or provided details of any sample size calculation used. The detailed score for each study in every item of the ARRIVE Guidelines is presented as Table 1S. The only study that evaluated human subjects was assessed using the PEDro scale and scored 5/10 according to this scale, which indicates a quality classified as “fair.” This study met criteria such as concealed sample allocation, baseline comparability (similar baseline between groups for main outcomes) and blinded subjects.

## Discussion

Recent studies have highlighted BDNF as an important neurotrophic factor involved in motor learning, recovery and neural rehabilitation after a stroke (7, 8, 36). The current review illustrates the consistencies and discrepancies in the literature regarding the response of BDNF concentrations following physical exercise training in subjects post-stroke or animals submitted to experimental CNS injury. It is worth mentioning that, although some investigations in humans have been performed recently, much of the understanding of changes in the BDNF concentration with exercise derives from animal studies. For example, only one study included in this review was completed in human subjects (19).

In general terms, aerobic exercise training appears to promote changes in central BDNF concentrations post-experimental stroke in animals, while central BDNF responses following non-aerobic exercise training in the animal model of stroke are still controversial. Most studies included in this review evaluated brain (local) BDNF concentrations in animal experimental designs. Treadmill training was the most common type of intervention among the studies and most of them showed an increase in BDNF concentration after this intervention. On the other hand, a few studies were performed to investigate the effect of non-aerobic training on BDNF concentration, such as reaching training (32, 34) and constraint induced movement therapy (CIMT) (17, 18), and the results among these studies are in discordance. Besides the differences in intervention protocols, a considerable variety of methods used to measure the BDNF concentrations was observed among the studies. Most of the methods used to measure BDNF concentration (e.g., ELISA, PCR, Western Blotting, Immunohistochemistry) are sensitive techniques, although they provide specific information regarding BDNF concentration. For example, ELISA provides an accurate protein quantitation in homogenates, while Western Blotting is not so accurate for quantitation, but provides information about the possible different molecular variants (e.g., pro-BDNF vs. BNDF mature). These differences should be taken into account when comparing the results of the studies.

### Aerobic training

Aerobic exercise, such as treadmill training, is continually used in rehabilitation due to its effectiveness in improving function, mobility and cardiovascular fitness in patients with chronic stroke (37–39). Generally, this review shows that aerobic exercise is able to promote changes in central BDNF concentrations in animal models of stroke. These results are in accordance with a previous systematic review showing that forced exercise at moderate to high intensity increases BDNF in multiple brain regions in animal models of stroke (8). However, the brain area where these changes occur varied among the studies included in the current review (14–16, 21–28). Both ipsilateral and/or contralateral changes in BDNF concentrations were found in the studies, and the most common brain areas evaluated were hippocampus, striatum and motor cortex. For example, Quirie et al. (25) evaluated the effect of treadmill training on central BDNF concentrations and, as compared to the striatum and the hippocampus, the cortex showed the greatest increase in BDNF following training. Other authors, however, observed a stronger increase in BDNF concentrations in the hippocampus following treadmill training (28). The different findings might be related to the time after the end of training session at which the BDNF was measured. It seems that when measured immediately after the last treatment session, the hippocampus shows higher BDNF levels. More studies are needed to confirm this hypothesis.

Training parameters such as the type of aerobic exercise, intensity and duration are also important factors that might affect BDNF concentration and motor function recovery (21–23, 28). Sun et al. (28) evaluated the effect of different treadmill training intensities on motor function recovery and neurorehabilitation, including the analysis of BDNF levels early after an experimental stroke in an animal model. Briefly, authors identified that training with gradually increased intensity achieved higher BDNF levels and better recovery, although an increase in BDNF was also observed following a low or high intensity exercise (see Table 1 for protocol details). Furthermore, there is also evidence that lower intensity endurance exercise leads to a more prolonged upregulation in central BDNF concentrations (up to 2 h) when compared to short periods of intense exercise (22). Such findings suggest that the brain BDNF response may be intensity sensitive, which is in agreement with previous findings from neurologically intact human subjects (40, 41). However, conclusions regarding the most effective aerobic training parameters for increasing BDNF concentrations are still limited post-stroke, given the large heterogeneity across studies available in the literature.

Only one study evaluated systemic BDNF concentration after exercise training (19). El-Tamawy et al. (19) investigated the effect of aerobic exercise (bicycle ergometer) on the serum BDNF concentration of post-stroke subjects (19). Although the heterogeneity of the sample was considerable (time post-stroke varying from 3 to 18 months), serum BDNF concentration increased significantly after 8 weeks of bicycle ergometer training, compared to pre-training and compared to the group that was submitted only to conventional physical therapy. Although this study does show relevant information regarding the effect of aerobic exercise training on BDNF concentrations post stroke in humans, the intensity of training was not described in the study. Previous studies in neurologically intact human subjects provide evidence that aerobic exercise training increases BDNF levels and the magnitude of BDNF increase seems to be exercise intensity dependent (13, 40, 42). Therefore, the training parameters and effort levels should be addressed in future studies in subjects post-stroke to identify the most appropriate training parameters that would result in increased levels of BDNF.

### Functional training

The findings arising from studies that evaluated the effect of functional training on BDNF levels are controversial. Among six studies, three showed an increase in central BDNF concentration following functional training (17, 32, 35), while the other three observed no change (18, 33, 34). A possible reason for this inconsistency might be related to the type of functional training and differences in training parameters. The time post-experimental stroke when the BDNF concentrations were evaluated varied among studies as well, which could be another source of disagreement between results. Studies in neurologically intact humans have also shown discrepancies in the effect of non-aerobic training, such as strength training, on BDNF levels (42, 43, 44). Accordingly, it may not be surprising that the results of non-aerobic training studies (functional training) post-stroke and its effects on BDNF concentrations are inconsistent.

In addition to examining the effects of aerobic exercise training on BDNF, studies in neurologically intact subjects have examined the effect of a short bout of aerobic exercise coupled with functional task practice (45, 46). These studies suggest that aerobic exercise may in fact “prime” the brain to learn subsequent motor tasks (45, 46). Although the mechanisms underlying this “priming” phenomenon are not completely clear, the upregulation of BDNF within the CNS following aerobic exercise could be a contributing factor that facilitates motor learning (10). Among all studies included in the current review, however, only one evaluated the effect of aerobic training (running) performed immediately before a reaching task on functional recovery and BDNF levels post-stroke (experimental model). There was a trend for slightly higher levels of central BDNF in both groups that performed an isolated reaching task or running immediately before the reaching task when compared to control, but no significant differences were observed. Therefore, further investigation is needed in order to clarify the role of BDNF in aerobic exercise applied as a “priming” before or after motor learning task post-stroke.

### Future directions

All but one study included in this review were performed using animals. Although these studies conducted on animals provide important information, it is unclear whether similar responses would be observed in humans. Further studies in humans post-stroke are needed to identify the most appropriate protocol of treatment to increase BDNF concentration and its correlation with motor recovery. This is, however, complicated because it is unclear whether or not BDNF can cross the human blood-brain barrier, which limits the interpretation of systemic BDNF measures in humans. In addition, more evidence is needed regarding systemic BDNF responses to exercise comparing the different phases of post-stroke recovery, such as acute, sub-acute and chronic phases.

## Conclusion

The results of the current systematic review highlight that aerobic exercise can promote changes in central BDNF concentrations in animal models of stroke, while BDNF responses following non-aerobic exercises, such as reaching training and CIMT, are still controversial.

## Author contributions

CA, LG-S, MS-C, and GS conducted literature searches, selected the studies and wrote the manuscript. CA, LG-S, MS-C, GS, DR, and TR contributed to the editing of the manuscript, final version and approval.

### Conflict of interest statement

The authors declare that the research was conducted in the absence of any commercial or financial relationships that could be construed as a potential conflict of interest.

## Supplementary material

Table 1S Methodological quality of studies included in this review scored according to ARRIVE Guidelines.
