# Supplementary material for: Post-stroke BDNF Concentration Changes Following Physical Exercise: A Systematic Review
Source: Front Neurol. 2018 Aug 28;9:637. doi: 10.3389/fneur.2018.00637 (PMC6121011; doi:10.3389/fneur.2018.00637)
Supplement: Table 1S — Methodological quality of studies included in this review scored according to ARRIVE Guidelines. [file Table_1.DOCX]

| AUTHOR, YEAR | Title | Abstract | Introduction - Background | Introduction -Objectives | Methods - Ethical statement | Methods - Study design | Methods - Experimental procedures | Methods - Experimental animals | Methods - Housing and husbandry | Methods - Sample size | Methods - Allocating animals to experimental groups | Methods - Experimental outcomes | Methods - Statistical methods | Results - Baseline data | Results - Numbers analysed | Results - Outcomes and estimation | Results - Adverse events | Discussion - Interpretation/scientific  implications | Discussion -Generalisability/translation | Discussion -Funding | *Total* |
| --- | --- | --- | --- | --- | --- | --- | --- | --- | --- | --- | --- | --- | --- | --- | --- | --- | --- | --- | --- | --- | --- |
| *Aerobic exercise* |  |  |  |  |  |  |  |  |  |  |  |  |  |  |  |  |  |  |  |  |  |
| Kim et al., 2005 | X | X |  | X | X |  |  | X |  |  | X | X | X |  | X | X |  | X |  | X | **12/20** |
| Ploughman et al., 2005 | X | X |  | X | X |  |  | X | X |  |  | X | X |  |  | X |  | X | X | X | **12/20** |
| Ploughman et al., 2007 | X | X |  |  | X |  |  | X | X |  |  | X | X |  | X | X |  | X | X | X | **12/20** |
| Ke et al., 2011 | X | X |  | X | X |  |  | X |  |  |  |  | X | X | X | X |  | X |  | X | **11/20** |
| Chen et al., 2012 | X | X |  | X | X |  |  | X | X |  |  |  |  |  |  |  |  | X |  | X | **8/20** |
| Quirie et al., 2012 | X | X | X | X | X |  | X | X | X |  | X | X | X |  |  | X |  | X |  |  | **13/20** |
| Zhang et al., 2013 | X | X |  | X | X | X | X | X |  |  | X | X | X |  | X | X |  | X | X |  | **14/20** |
| Banoujaafar et al., 2014 | X | X |  | X | X |  | X | X | X |  | X | X |  |  |  | X |  | X | X |  | **12/20** |
| Lan et al., 2014 | X | X |  | X | X |  | X | X | X |  | X | X | X |  |  | X |  | X |  |  | **12/20** |
| Seo et al. 2014 | X | X |  |  | X |  |  | X |  |  | X |  | X |  | X | X |  | X | X | X | **11/20** |
| Sun et al., 2014 | X | X |  |  | X |  | X | X |  |  |  |  | X |  | X | X |  |  | X | X | **10/20** |
| Ahn et al., 2016 | X | X | X | X | X |  | X | X |  |  |  | X | X |  | X | X |  | X | X | X | **14/20** |
| Himi et al., 2016 | X | X | X | X | X |  |  | X | X |  |  | X | X | X | X | X |  | X | X | X | **15/20** |
| Takamatsu et al., 2016 | X | X |  |  | X |  |  | X |  |  |  |  | X |  | X | X |  | X | X | X | **10/20** |
|  |  |  |  |  |  |  |  |  |  |  |  |  |  |  |  |  |  |  |  |  |  |
| *Functional training* |  |  |  |  |  |  |  |  |  |  |  |  |  |  |  |  |  |  |  |  |  |
| Livingstone-Thomas et al., 2013 | X | X | X | X | X |  | X | X | X |  | X | X | X |  |  | X |  | X | X | X | **15/20** |
| Yong et al., 2014 | X | X |  | X | X |  | X | X | X |  |  | X | X |  |  | X |  | X |  |  | **11/20** |
| Ishida et al., 2015 | X | X |  | X | X |  | X | X | X |  |  | X | X |  | X | X |  | X |  | X | **13/20** |
| Takamoshi et al., 2016 | X | X |  | X | X |  | X | X | X |  |  | X | X |  |  | X |  | X |  | X | **12/20** |
|  |  |  |  |  |  |  |  |  |  |  |  |  |  |  |  |  |  |  |  |  |  |
| *Aerobic exercise / Functional training* |  |  |  |  |  |  |  |  |  |  |  |  |  |  |  |  |  |  |  |  |  |
| Ploughman et al., 2007 | X | X |  | X | X |  | X | X | X |  |  | X | X |  |  | X |  | X | X | X | **13/20** |
| Yong et al., 2017 | X | X |  | X | X |  |  | X |  |  |  |  |  |  |  | X |  | X |  | X | **8/20** |
